# Comparison of Health Care Worker Satisfaction Before vs After Implementation of a Communication and Optimal Resolution Program in Acute Care Hospitals

**DOI:** 10.1001/jamanetworkopen.2023.2302

**Published:** 2023-03-09

**Authors:** Andrew I. Friedson, Abigail Humphreys, Florence LeCraw, Barbara Pelletreau, Priscilla Vierra, Thomas A. Mroz

**Affiliations:** 1 Department of Economics, University of Colorado, Denver; 2 Northside Healthcare System, Atlanta, Georgia; 3 Andrew Young School of Policy Studies, Georgia State University, Atlanta; 4 CommonSpirit Health, Chicago, Illinois; 5 Federal Reserve Bank of Atlanta, Atlanta, Georgia

## Abstract

This case-control study investigates the association between a communication and optimal resolution program to address unexpected adverse patient outcomes and measures of health care worker satisfaction.

## Introduction

Considerable health care professional (HCP) burnout happens due to work culture in medical settings, often leading to HCPs leaving the health care sector. This can lead to lower access to care and increased risk of medical error. Factors that may contribute to burnout include low satisfaction with one’s work and lack of support by health care leadership.^1,2^

We investigate the association between a communication and optimal resolution (CANDOR) program and measures of HCP satisfaction. CANDOR is used when a patient experiences an unexpected adverse outcome, typically replacing the common “deny and defend” approach. CANDOR includes an explanation, an apology if appropriate, discussion of prevention of recurrence of the error, proactive compensation, and peer support for HCPs whose patients experience harm. CANDOR is related to liability and patient safety outcomes (as is honesty in general), but no studies have examined CANDOR’s association with HCP satisfaction.^3,4^ We hypothesize that CANDOR is positively associated with HCP satisfaction.

## Methods

This case-control study was approved by the CommonSpirit Health (CSH) institutional review board. The requirement for informed consent was waived because data used were aggregate responses and individually identifiable data were never obtained. This study followed the Strengthening the Reporting of Observational Studies in Epidemiology (STROBE) reporting guideline. For this study, we examined annual employee surveys in CSH system’s acute care hospitals from 2016 to 2019. We recorded the date the first case closed under CANDOR as each hospital’s initiation date of CANDOR. These dates signified the time when many hospital HCPs could become aware of CANDOR implementation.

We applied a difference-in-differences analysis, comparing annual mean survey responses before and after CANDOR initiation dates with those at hospitals that had not yet implemented CANDOR. This is a form of case-control study, where cases and controls are determined by whether a facility already had initiated CANDOR, a somewhat random occurrence.

Responses studied included measures of workplace satisfaction, perceptions of safety, and satisfaction with leadership. Survey participants rated how much they agree with each statement on a 5-point Likert scale (we reversed the scale for questions with a negative connotation; higher numbers always indicate a better, more positive feeling). The eAppendix in Supplement 1 provides a list of studied responses.

The estimation applied a novel technique that corrects for bias due to differential timing of events^5^ to calculate the association between CANDOR implementation at facilities and the outcomes of worker satisfaction surveys. Associations were tested using a *t* test nested within regressions, and statistical significance is assessed at 2-sided *P* < .05. Data were analyzed from August 2022 to January 2023.

## Results

There were 56 acute care hospitals with survey data for all 4 years, and 19 had their first event handled via CANDOR during the study period. There was a mean (SD) of 1130 (884) annual survey responses before CANDOR and 1131 (995) responses after CANDOR.

We did not find any statistically significant association between CANDOR implementation and responses concerning HCP’s perceptions of hospital safety (Table 1). For example, the estimate of the association between CANDOR implementation and Likert ratings of “I would feel safe being treated here as a patient,” had a trivial point estimate (average treatment on treated, −0.014; 95% CI, −0.078 to 0.050) and was not statistically significant (*P* = .66).

**Table 1.** Changes in Survey Responses on Perception of Safety After First Communication and Optimal Resolution Program Event

Response	ATT (95% CI)^a^	*P* value	Observations	Preevent mean outcome
In this work setting, it is difficult to discuss errors^b^	−0.027 (−0.093 to 0.040)	.44	206	3.439
Medical errors are handled appropriately in this work setting	0.034 (−0.021 to 0.088)	.23	206	3.941
I would feel safe being treated here as a patient	−0.014 (−0.078 to 0.050)	.67	206	3.926
The culture in this work setting makes it easy to learn from the errors of others	0.022 (−0.036 to 0.079)	.46	206	3.656

Abbreviation: ATT, average treatment on treated.

^a^ Regressions were estimated using the model for difference-in-differences in multiple time periods. Outcomes are modeled with weighted least squares, and treatment is modeled with inverse probability tilting.

^b^ Reverse coded, meaning that higher values are more desirable responses.

Most measures of workplace satisfaction and of trust in leadership were statistically significantly associated with a first CANDOR event (Table 2). For example, the estimated association between a CANDOR event and Likert ratings of “I would work for this organization three years from now,” had a point estimate of 0.057 (95% CI, 0.007 to 0.106; *P* = .03), a 1.4% increase from the baseline mean response.

**Table 2.** Changes in Survey Responses on Worker Satisfaction After First Communication and Optimal Resolution Program Event

Response	ATT (95% CI)^a^	*P* value	Observations	Preevent mean outcome
I would recommend a location within this organization	0.063 (0.002 to 0.125)	.04	224	3.981
I would recommend this organization as a great place to work	0.073 (0.004 to 0.128)	.04	224	3.393
We demonstrate our core value of dignity by how we listen	0.044 (−0.007 to 0.094)	.09	224	4.061
Management understands how the work contributes to the patient experience	0.065 (0.001 to 0.128)	.046	224	3.853
I would work for this organization three years from now	0.057 (0.007 to 0.106)	.03	224	4.220
Senior leadership is leading us in the right direction	0.081 (−0.009 to 0.172)	.08	224	3.624
Management creates an environment of trust	0.080 (0.009 to 0.151)	.03	224	3.757
Management holds all employees to the same standard	0.103 (0.021 to 0.185)	.01	168	3.733

Abbreviation: ATT, average treatment on treated.

^a^ Regressions were estimated using the model for difference-in-differences in multiple time periods. Outcomes are modeled with weighted least squares, and treatment is modeled with inverse probability tilting.

## Discussion

In this case-control study in a single hospital system, CANDOR was associated with increases in most measures of HCP workplace satisfaction and HCP satisfaction with hospital leadership, which are both correlated with burnout and turnover.^1,2^ There was no association between CANDOR and perceptions of safety. This study cannot observe or control for the composition of participants in the satisfaction surveys. Findings do not necessarily generalize to other related policies or institutional contexts.

## Notes

Supplement 1. **eAppendix. **Detailed Description of Data

Supplement 2.
Data Sharing Statement
