# Supplementary material for: Comparison of Health Care Worker Satisfaction Before vs After Implementation of a Communication and Optimal Resolution Program in Acute Care Hospitals
Source: JAMA Netw Open. 2023 Mar 9;6(3):e232302. doi: 10.1001/jamanetworkopen.2023.2302 (PMC9999242; doi:10.1001/jamanetworkopen.2023.2302)
Supplement: Supplement 1. — eAppendix. Detailed Description of Data [file jamanetwopen-e232302-s001.pdf]

Supplemental Online Content

Friedson AI, Humphreys A, LeCraw F, Pelletreau B, Vierra P, Mroz TA. Comparison of health care worker satisfaction before vs after implementation of a communication and optimal resolution program in acute care hospitals. *JAMA Netw Open*. 2023;6(3):e232302. doi:10.1001/jamanetworkopen.2023.2302

eAppendix. Detailed Description of Data

This supplemental material has been provided by the authors to give readers additional information about their work.

© 2023 Friedson AI et al. *JAMA Network Open.*

eAppendix. Detailed Description of Data

Data come from employee surveys administered annually in all CommonSpirit facilities. Each survey contains multiple questions about various parts of the employee experience that are answered on a 1 to 5 Likert Scale with 1 being the strongest disagreement and 5 being the strongest agreement. We used the following statements as our outcome variables of interest.

eTable 1. Survey Variables

**Statement Text**
In this work setting, it is difficult to discuss errors*
Medical errors are handled appropriately in this work setting
I would feel safe being treated here as a patient
The culture in this work setting makes it easy to learn from the errors of others
I would recommend a location within this organization
I would work for this organization three years from now
I would recommend this organization as a great place to work
Senior leadership is leading us in the right direction
We demonstrate our core value of dignity by how we listen
Management creates an environment of trust
Management understands how the work contributes to the patient experience
Management holds all employees to the same standard

* Reverse coded after data collection such that 5 refers to strong disagreement

Data also come from internal records which track all adverse patient events handled through Communication and Optimal Resolution (CANDOR) protocols. For each hospital, we assign it the date that the first CANDOR event was closed (i.e. completed).

<u>Detailed Description of Differences-in-Differences Model</u>

Typically, a difference-in-differences model compares the difference in the outcome of interest before and after a treatment date between a treatment group to that in a control group. The first difference is thus over time, and the second difference is between the two groups. The key assumptions for validity are that the treatment and control groups share a common trend prior to treatment, and that no other event occurs simultaneously that impacts the groups differentially.

The model can be adapted to contain multiple different treated entities, in our case the 19 acute care hospitals (of a total of 56) that had a CANDOR event close during the timeframe. Some difficulty arises when the treated entities are treated at different times, as the standard "two-way fixed effects" estimator for a difference-in-differences analysis can make comparisons that violate the assumptions of the model, in part because a group that is treated later in time may be

© 2023 Friedson AI et al. *JAMA Network Open.*

used as a control for one that is treated earlier in time (see Goodman-Bacon (2021) for a deeper discussion of these issues).

One way to sidestep this issue is to restrict to "valid" comparisons between eventually-treated units and never-treated units. This is what is done in the method proposed by Callaway and Sant'Anna (2021), which we implement for our estimation. Practically, we conduct the estimation using the "csdid" command in STATA 17, which estimates the model proposed in Callaway and Sant'Anna (2021). The Callaway and Sant'Anna (2021) estimator is a refinement of the doubly robust differences-in-differences estimator from Sant'Anna and Zhao (2020). This method calculates the "average effect of treatment on the treated" (ATT) for each valid comparison between a treated group and a control group, the ATTs are then averaged into an overall ATT, which is the difference-in-differences estimator for the overall analysis, albeit expunged of bias stemming from invalid comparisons.

References

Callaway, B. and Sant'Anna, P.H., 2021. Difference-in-differences with multiple time periods. Journal of Econometrics, 225(2), pp.200-230.

Goodman-Bacon, A., 2021. Difference-in-differences with variation in treatment timing. Journal of Econometrics, 225(2), pp.254-277.

Sant'Anna, P.H. and Zhao, J., 2020. Doubly robust difference-in-differences estimators. Journal of Econometrics, 219(1), pp.101-122.

© 2023 Friedson AI et al. *JAMA Network Open.*
