# Supplementary material for: Comparison of Health Care Worker Satisfaction Before vs After Implementation of a Communication and Optimal Resolution Program in Acute Care Hospitals
Source: JAMA Netw Open. 2023 Mar 9;6(3):e232302. doi: 10.1001/jamanetworkopen.2023.2302 (PMC9999242; doi:10.1001/jamanetworkopen.2023.2302)
Supplement: Supplement 2. — Data Sharing Statement [file jamanetwopen-e232302-s002.pdf]

Data Sharing Statement

Friedson. Comparison of Health Care Worker Satisfaction Before vs After Implementation of a Communication and Optimal Resolution Program in Acute Care Hospitals. *JAMA Netw Open*. Published March 09, 2023. doi:10.1001/jamanetworkopen.2023.2302

Data
Data available: No

Additional Information
Explanation for why data not available: Data are proprietary to CommonSpirit Health
